# Early malignant syphilis*

**DOI:** 10.1590/abd1806-4841.20164491

**Published:** 2016

**Authors:** Yara Martins Ortigosa, Paulo Salomão Bendazzoli, Angela Marques Barbosa, Luciena Cegatto Martins Ortigosa

**Affiliations:** 1 Universidade Estadual Paulista "Júlio de Mesquita Filho" (Unesp) – São Paulo (SP), Brazil; 2 Santa Casa de Marília – Marília (SP), Brazil; 3 Universidade do Oeste Paulista (Unoeste) – Presidente Prudente (SP), Brazil; 4 Universidade de São Paulo (USP) – São Paulo (SP), Brazil

**Keywords:** Diabetes mellitus, Syphilis, Treponema pallidum

## Abstract

Early malignant syphilis is a rare and severe variant of secondary syphilis. It is clinically characterized by lesions, which can suppurate and be accompanied by systemic symptoms such as high fever, asthenia, myalgia, and torpor state. We report a diabetic patient with characteristic features of the disease showing favorable evolution of the lesions after appropriate treatment.

## INTRODUCTION

Early malignant syphilis (EMS) is a rare and ulcerative form of secondary syphilis.^1,2^ Its name derives from the similarity of the lesions with some cutaneous neoplasias.^3,4^ It is characterized by ulcerated papules, plaques, and necrotic nodules, often with a rupioid appearance.^5,6^

Serology generally shows high titers, positive inflammatory tests, and abnormal transaminases.^4^

Patients are usually impaired, in poor health, and with some kind of immunodeficiency. The disease also affects pregnant women, nursing mothers, and alcoholics.^7,8^

We report a case of EMS in a diabetic patient in order to draw attention to the diagnosis of the disease and its association not only with HIV, but also with other types of immunosuppression – in our case, diabetes mellitus (DM).

## CASE REPORT

We report a 53-year-old white widow patient from Presidente Prudente (SP - Brazil) who presented with diabetes mellitus (DM) for 7 years treated with sitagliptin and metformin hydrochloride. Twenty days prior to admission, she noticed a wound in the vulvar region, which resolved spontaneously. After that, she experienced poor overall condition and was affected by disseminated lesions, fever (not verified), and myalgia.

**Figure 1:**
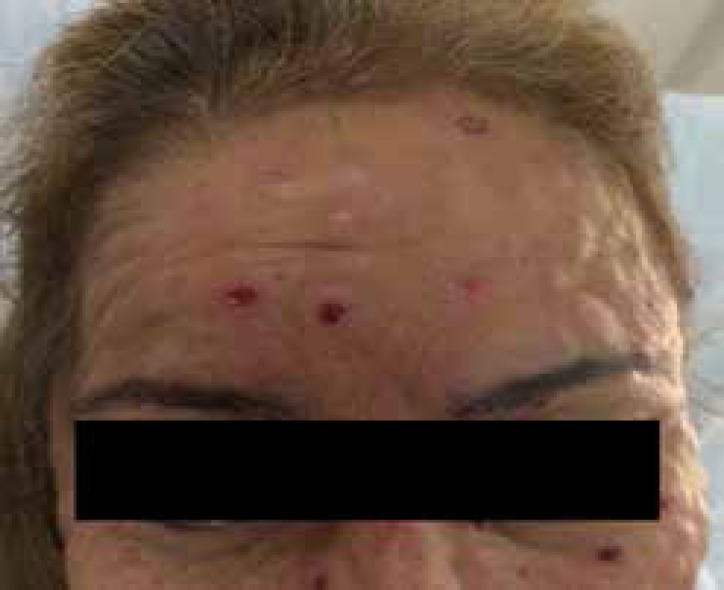
Dissemina - ted papules and nodules; some ulcerated lesions with well-defined edges and base with granulation tissue; some with rupioid crusts on the face

**Figure 2:**
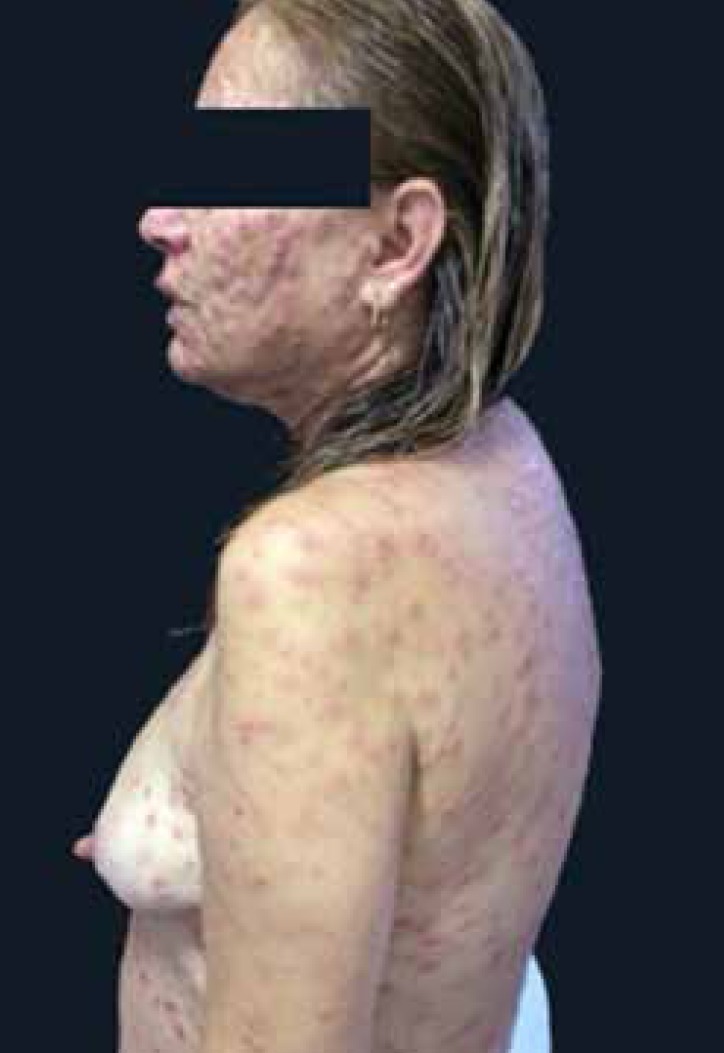
Purplish erythematous papules nodules scattered on the trunk

**Figure 3:**
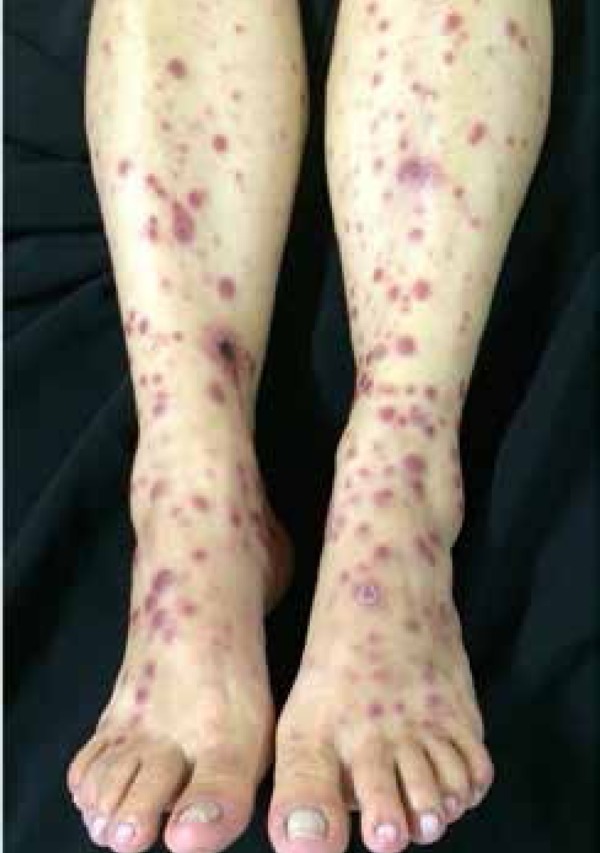
Erythematous papules and nodules and wines and some ulcerated lesions on the lower limbs

Dermatological examination revealed infiltrating erythematous-violaceous papules and nodules, some ulcerated, 0.2-0.5 cm in diameter, with clear limits and regular contours symmetrically spread on the head, chest, and limbs (Figures 1-3). We also observed erythematous palmoplantar papules and palpable painful lymph nodes in the cervical, axillary, and inguinal regions.

Complementary tests showed normal CBC, negative ANA, ANCA, RNP tests, negative toxoplasmosis, negative hepatitis B and C, and HIV, blood sugar values of 249 mg/dL, ESR of 45 mm/ml, reactive VDRL (1/8), and positive FTA-ABS. The CSF analysis was within the normal range.

**Figure 4:**
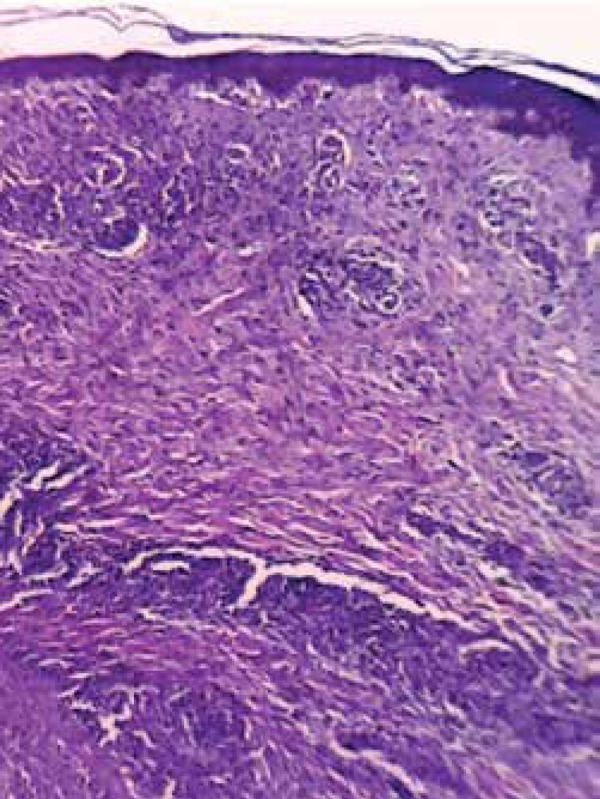
Histopathological examination showing dermal, superficial , and deep inflammatory infiltrate (HE, 40x)

**Figure 5:**
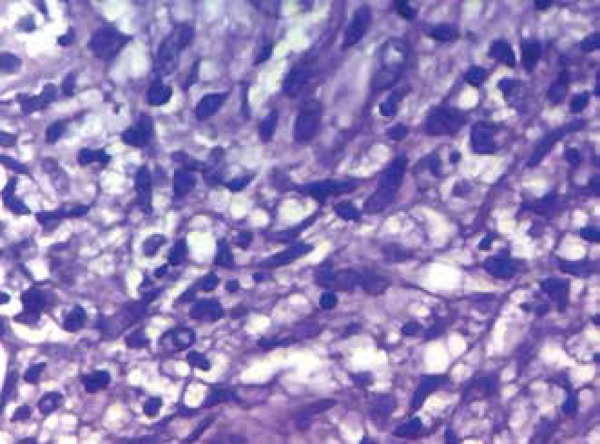
Detail of one of the areas in which the inflammatory infiltrate outlines loose granulomas (HE, 400x)

The anatomopathological examination of the abdominal skin lesion revealed interface dermatitis with a loose cluster of non-caseating granulomas, presence of plasma cells with rare occurrence of eosinophils, and endothelial swelling with red blood cell extravasation (Figures 4 and 5).

Considering the clinical picture, we opted for treatment with penicillin G benzathine (three doses of 2,400,000 units injected with a one-week interval) and prednisone (60 mg daily) because of the intense inflammatory process.

Her partner’s complementary tests showed reactive VDRL (1/256), passive hemagglutination, and positive FTA-ABS.

## DISCUSSION

Early malignant syphilis (EMS) was described by Bazin in 1859 and Dubuc in 1864 as a nodular variant of secondary syphilis with aggressive development.^6,8-10^ Its diagnosis was common in the seventeenth century, but its incidence has subsequently declined.^4^ Years before the discovery of penicillin, in times of war and famine, EMS was observed in cachectic patients with tuberculosis.^6^ Its unusual clinical manifestation is caused by poor health conditions, malnutrition and inappropriate use of immunosuppressants or antibiotics. Currently, coinfection with HIV is the most frequent cause of the disease.^7,8^

The lesions are characterized by erythematous-violaceous or reddish-coppery ulcerated papules, nodules, or blisters. They can progress to necrosis giving rise to rupioid crusts that resemble an oyster shell.^2^ In some cases, they form small ulcers with well-defined edges, covered with purulent secretion without perilesional inflammatory reaction.^4^ These lesions follow the chancre formation or arise a few months after it.^5^ There may be mucosa involvement, and prodromes such as headache, arthralgia, and myalgia are common. Concomitant gastrointestinal symptoms – diarrhea and vomiting – are described, as well as hepatosplenomegaly and lymphadenopathy.^5^ The evolution of the disease leads to the impairment of general condition and a lethal outcome is possible if appropriate therapy is not administered.^6^

The lesions are caused by medium-sized-vessel vasculitis affecting the dermis.^1,10^ Qualitative or functional defects (or both) on the humoral and cellular responses are probably involved in the pathogenesis of the disease.^9^

Theories indicate that changes to this exuberant clinical picture is due to the immune depressed state of the individual, to more virulent strains of Treponema pallidum, or to an exuberant immune response of the patient. Supporting the latter theory, a severe form of syphilis associated with chronic diseases – such as malaria and tuberculosis – was reported in the nineteenth century.^6^

The EMS diagnostic criteria described by Fischer *et al.* include strongly positive results for syphilis, Jarisch-Herxheimer reaction, and response to appropriate antibiotic treatment.^9,10^ The present case showed excellent response to treatment and compatible pathology, but low-titer of specific antibody. Perhaps the excess antibodies in the tested serum caused this result (prozone phenomenon). We observed no Herxheimer reaction, probably because of the concomitant use of corticoids.

Differential diagnosis of EMS includes ulcerative pyoderma, chronic pityriasis lichenoid, acute varicelliform versicolor, papulosis lymphomatoid, lymphoma, leprosy, and necrotizing generalized herpes zoster, among other diseases.^6^ Pathological studies showing obliterative medium-sized-vessel vasculitis and plasma cells infiltrates in the dermis can be valuable tools in case of a challenging diagnosis.^1,2,10^

There is no special recommended treatment for EMS. Although the current treatment for secondary syphilis consists of two penicillin G benzathine injections at a dose of 2,400,000 IU at weekly intervals, in our case we chose the total dose of 7,200,000 IU, as some authors recommend increasing the dose in case of HIV coinfection or in cases of immunosuppression.^1-4,6,8,10^ For resistant cases or relapses, prolonged therapy with high doses of penicillin is suggested.^6^

The occurrence of the EMS in case of DM-induced immunosuppression, as shown in the present case, is extremely rare. A few cases of the disease are reported, mostly associated with HIV.^5,10^ Our literature review showed only one similar case reported in Germany.^6^

We reported a diabetic patient with exuberant lesions, which shows that we must consider EMS not only in association with HIV, but also in cases where the weakened state of the patient can lead to immunodeficiency. This atypical presentation of the disease would make diagnosis challenging.

Syphilis, despite being described since the dawn of humanity, is a still relatively common disease in our midst. Thus, by presenting varied clinical manifestations and mimicking several dermatoses, it should always be included in differential diagnoses.
